# Pseudoprogression manifesting as recurrent ascites with anti-PD-1 immunotherapy in urothelial bladder cancer

**DOI:** 10.1186/s40425-018-0334-x

**Published:** 2018-04-04

**Authors:** Randy F. Sweis, Yuanyuan Zha, Lomax Pass, Brian Heiss, Tara Chongsuwat, Jason J. Luke, Thomas F. Gajewski, Russell Szmulewitz

**Affiliations:** 1 Department of Medicine, Section of Hematology/Oncology, Comprehensive Cancer Center, University of Chicago Medicine, 5841 S. South Maryland Avenue, MC 2115, Chicago, IL 60637 USA; 2 Department of Pathology, University of Chicago, 5841 S. Maryland Avenue, MC 2115, Chicago, IL 60637 USA; 3 University of Chicago, 5841 S. Maryland Avenue, MC 2115, Chicago, IL 60637 USA

**Keywords:** Immunotherapy, Pseudoprogression, PD-1, Bladder urothelial cancer, Immune checkpoint, Pembrolizumab

## Abstract

**Background:**

Immunotherapies targeting the PD-1 checkpoint pathway have recently gained regulatory approval in numerous cancer types. With the widespread use of immune checkpoint therapies, varying patterns of responses and immune-related adverse events are being observed.

**Case Presentation:**

In this case, we highlight a patient who developed recurrent, large-volume ascites, while simultaneously having a 49% reduction in peritoneal tumor lesion size by RECIST criteria. Sampling of the fluid revealed high levels of IL-6 and IL-15. Cytology revealed no malignant cells on 4 separate paracenteses over a period of 6 weeks. Cell counts revealed that 45% of cells were lymphocytes, and further analysis was performed by fluorescence-activated cell sorting (FACS). The majority of lymphocytes were CD8^+^, of which 78% were PD-1^+^ and 43% were HLA-DR^+^ indicating an activated phenotype.

**Conclusions:**

In summary, treatment with anti-PD-1 therapy may result in pseudoprogression manifested by ascitic fluid accumulation due to the influx of activated T cells. Since worsening of ascites is typically associated with disease progression, it is important to consider the possibility of pesudoprogression in such patients undergoing therapy with immune checkpoint inhibitors.

**Electronic supplementary material:**

The online version of this article (10.1186/s40425-018-0334-x) contains supplementary material, which is available to authorized users.

## Background

Antibodies targeting negative regulation of immune cells, known as immune checkpoint inhibitors, have dramatically impacted the therapeutic landscape for numerous cancers. In bladder cancer, five immunotherapies targeting the PD-1 pathway were approved between 2015 and 2016 after a period of decades without any new drug approvals [1–6]. Disinhibiting negative regulation on immune cells is associated with a distinct pattern of toxicities and is also associated with unique radiographic patterns of response. Given the rapid and widespread uptake in the clinical use of immune checkpoint inhibitors, rarer toxicities and atypical clinical manifestations of responses are now being observed and reported. Pseudoprogression, for instance, is a phenomenon that is manifested by apparent progression on imaging followed by subsequent regression in tumor size [7–11]. In melanoma, 28% of patients treated beyond progression with the anti-PD1 inhibitor nivolumab had subsequent responses with greater than 30% reduction in target lesion size. Reports analyzing tumor tissue in this setting have reported the influx of T lymphocytes and other immune cells [11]. Pseudoprogression is an important phenomenon to recognize and understand, since it may result in inappropriately discontinuing therapy in a patient who may actually be responding favorably. This concern has even led to the development of a distinct set of response criteria that account for pseudoprogression, in contrast to traditional methodology using Response Evaluation Criteria In Solid Tumors (RECIST) [12–14].

Pseudoprogression has also been noted to manifest not only radiographically, but also through clinical findings. Recently, two cases have been reported illustrating the development of pleural and pericardial effusions in patients with tumor regression after anti-PD-1 therapy with nivolumab [15]. Interestingly, analysis of the pericardial and pleural fluid showed 5% and 30% lymphocytes in those cases, respectively. To our knowledge, the development of ascites as a manifestation of pseudoprogresison has not been reported. Herein we highlight a case where a patient developed large-volume recurrent ascites with concurrent regression of peritoneal metastasis on imaging indicative of response.

## Case presentation

A 61-year-old woman developed hematuria and underwent cystoscopy revealing a large tumor in the posterolateral bladder wall. Biopsy revealed poorly differentiated muscle-invasive urothelial carcinoma. Immunohistochemical stains were positive for CK-7 and GATA-3, and negative for CK-20. She underwent two cycles of neoadjuvant chemotherapy with gemcitabine and cisplatin before treatment was discontinued due to severe neutropenia. She then elected for external beam radiation for 9 weeks without concurrent chemotherapy. PET-CT scan imaging showed a good response without any identified residual or recurrent bladder masses or lymphadenopathy. Six months later a residual tumor in the bladder was noted on cystoscopy. CT abdomen and pelvis showed a recurrent mass in the bladder with likely invasion into the vaginal cuff, an enlarged para-aortic nodule, and two peritoneal nodules. Surgery was not recommended, and she began second-line chemotherapy with pemetrexed. Shortly after one cycle, her performance status declined, she developed rectal bleeding, and was admitted to the hospital. Diagnostic workup with colonoscopy revealed angioectasias in the colon that were treated with argon plasma coagulation. Areas of erythematous, friable mucosa were noted, so she was diagnosed with radiation proctitis. She was evaluated by gastroenterology, treated supportively with sucralfate and mesalamine enemas, and discharged from the hospital. At her clinic follow up, pemetrexed was discontinued in favor of a clinical trial evaluating pembrolizumab in bladder cancer. Of note, she had a history of hepatitis C virus infection, which was previously cured with the combination of ledipasvir and sofosbuvir. Prior to starting pembrolizumab, polymerase chain reaction (PCR) testing confirmed an undetectable viral load. Her Child­Pugh score was 6 (Class A). She was started on pembrolizumab 200 mg every 3 weeks.

**Fig. 1:**
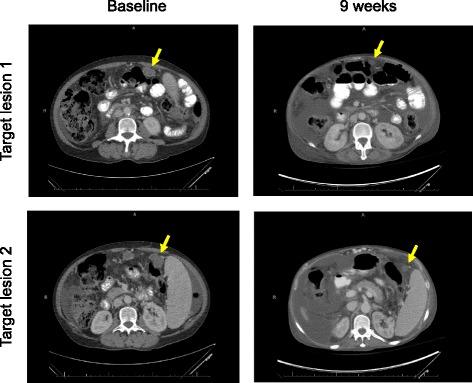
Baseline images (left) compared with 9 week CT scan (right) showing reduction in target lesion size with anti-PD-1 therapy

Prior to starting immunotherapy, she had no significant ascites on clinical exam or CT scans. Three weeks after starting anti-PD-1 therapy, she developed abdominal discomfort and early satiety, and was found to have large-volume ascites on clinical exam. She underwent diagnostic and palliative paracentesis, with 4500 ml of ascitic fluid drained. She had a marked improvement in her discomfort. The acities was considered a Grade 2 toxicity, which was not considered to be drug-related at the time. Cytology was performed and no malignant cells were identified in the fluid. The initial paracentesis fluid showed a serum-ascites albumin gradient (SAAG) > 1.5, total protein of 1.2 g/dL, and WBCs of 231/μL including 45% lymphocytes. Cytology only showed reactive cellular changes and inflammation. Over the next 6 weeks, she underwent 3 more diagnostic and palliative paracenteses and subsequent cytological analyses also showed inflammatory changes with no malignant cells. She never developed fever or leukocytosis. Liver function tests remained stable throughout this time period. After 3 cycles of therapy, her CT imaging showed a partial response per RECIST v1.1 with a 49% reduction in the size of her target lesions (Fig. 1).

**Fig. 2:**
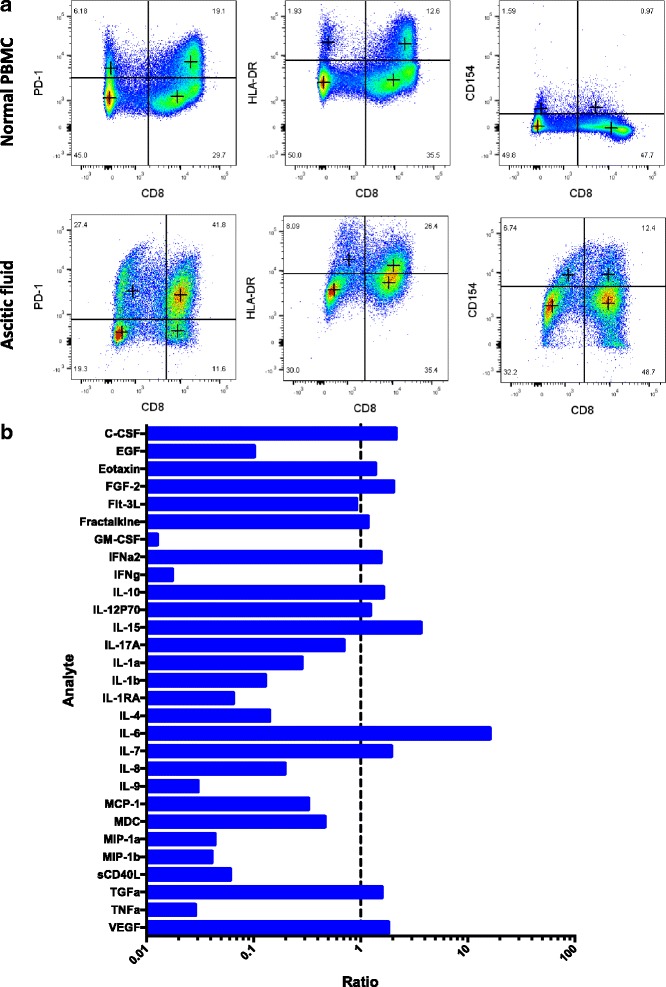
**a** FACS analysis of CD8+ T cell subsets for normal PBMC control (top panels) versus patient ascitic fluid (bottom panels). **b** Cytokine array showing analyte levels as ratios to healthy donor peripheral blood mononuclear cell (PBMC) sample controls stimulated with PMA and ionomycin (See Additional file 1: Table S1 for values)

Given the high percentage of lymphocytes in the peritoneal fluid, further characterization was performed by fluorescence-activated cell sorting (FACS). This analysis revealed that CD8^+^ lymphocytes comprised 53% of the CD45^+^CD3^+^ cells (Fig. 2a). The majority of CD8^+^ lymphocytes were PD-1^+^ (78%). Class II MHC (HLA-DR) and CD40 ligand (CD154) were expressed on 43% and 20% of CD8^+^ lymphocytes, respectively. All three markers were increased in comparison to a normal donor PBMC control, suggesting a locally high number of antigen-experienced, activated T cells. FoxP3^+^CD25^+^ Tregs were also detected as 17% of the CD45^+^CD3^+^CD4^+^ compartment. We also interrogated the fluid for the presences of cytokines using a multiplex cytokine assay for 38 analytes. The most significantly elevated factors were IL-6 and IL-15, which were measured more than 3-fold above healthy donor peripheral blood mononuclear cell (PBMC) sample controls (Fig. 2b and Additional file 1: Table S1).

Ultimately, the patient developed worsening symptoms and was hospitalized due to radiation proctitis and also a pulmonary infection. Despite the response anti-PD-1 therapy on imaging, her quality of life declined given these various comorbid conditions. She ultimately elected to discontinue all therapy and enrolled in hospice.

## Discussion

There is an emerging literature of unusual responses and toxicities to immune checkpoint therapy, including recurrent pleural effusions and cardiac tamponade [15]. Here we report a patient with metastatic urothelial cancer including peritoneal nodules who responded to anti-PD-1 therapy with pembrolizumab, but developed concurrent large-volume ascites. The ascites developed by the 3-week time point and persisted through the third cycle at 9 weeks, recurring despite 4 diagnostic and therapeutic paracenteses. The time course for the development of ascites in this case is consistent with that of classical imaging-based pseudoprogression and the recent reports of pleural and pericardial effusions [15, 16]. Typically, the recurrent accumulation of ascites in the setting of peritoneal carcinomatosis most commonly represents progression of disease. However, in this case the peritoneal fluid was sampled repeatedly to rule out the presence of malignant cells. The initial paracentesis showed a transudate with a SAAG > 1.5 and a total protein of 1.2 g/dL, which together are not as high as what is typical for peritoneal carcinomatosis. The cell counts demonstrated lymphocyte enrichment, and FACS analysis showed that the lymphocytes were antigen-experienced, activated CD8^+^ T cells.

Limited data are published regarding the T cell subpopulations found in cancer ascites. In one series, a phenotypic analysis showed the percentage of HLA-DR-expressing CD8^+^ T cells was higher in untreated ovarian cancer patients’ ascites (33%) versus non-malignant cirrhotic ascites (21%) [17]. Both of these percentages are lower than the 43% observed in the current case. Similarly, another ovarian cancer series identified activated T cells in pre-treatment ascitic fluid of patients at diagnosis [18]. However, in those cases the percentage of CD8^+^ T cells expressing PD-1 averaged less than 10%, which is markedly lower than the 78% observed in this case. Thus, a high percentage of PD-1^+^CD8^+^ and HLA-DR^+^CD8^+^ T cells might represent a biomarker for ascitic fluid pseudoprogression after immune checkpoint therapy.

It is possible that the development of ascites was secondary to a non-specific immune-related adverse event such as autoimmune peritonitis. However, the accumulation of antigen-experienced CD8^+^ T cells detected in the fluid with regression of peritoneal nodules suggests that the ascites was related to a specific anti-tumor immune response. Indeed, the presence of dysfunctional tumor antigen-specific T cells in ascitic fluid has been noted previously in the setting of progression when malignant cells were also detected [19]. The current case is unique in that tumor shrinkage was observed and no malignant cells were found on repeated sampling of the fluid. Further analysis of the fluid identified high levels of pro-inflammatory cytokines. IL-6 has various context-dependent functions and has been shown to promote anti-tumor immunity through recruitment and stimulation of T cells [20, 21]. Similarly, IL-15 is a cytokine that stimulates proliferation and activation of antigen-specific CD8^+^ T cells and is currently under investigation as a therapeutic [22–24]. All these findings taken together suggest that the patient developed ascites not due to worsening of peritoneal carcinomatosis as typically expected, but instead due to an expanded anti-tumor immune response following anti-PD-1 therapy. Her ascites was treated with therapeutic paracentesis only. Corticosteroids were not given, but in the future they might be considered for patients with similar findings in need of symptom palliation. On the other hand, if ascites in this context is indeed an on-target effect, then corticosteroid administration might not dampen the accumulation of ascitic fluid.

## Conclusion

In cancer patients treated with immunotherapy, pseudoprogression may manifest as recurrent ascites due to a rapid influx of activated T lymphocytes into the peritoneum. Since ascites can also be caused by disease progression, it is important to clinically distinguish between the two scenarios, so that treatment may be appropriately continued or stopped. In patients who develop ascites after immune checkpoint blockade, careful fluid analysis may provide additional evidence to help distinguish between potential etiologies. Cell counts indicating lymphocyte enrichment along with the absence of malignant cells on repeated sampling may suggest that an anti-tumor immune response is a more likely cause than disease progression. Clinical judgment remains key to determine the best course of action for an individual patient, since pseudoprogression is far less common in general than actual disease progression. With the recent adoption of immune checkpoint inhibitors in numerous cancer types, recognition and understanding of unusual patterns of response will be critical to optimize patient outcomes.

## Materials and methods

### Cell surface marker analysis

Lymphocyte subsets were analyzed by flow cytometry. The lymphocyte population was gated based on size and granularity, followed by CD45^+^CD3^+^ using PE-Cy5 anti-CD45 and PB anti-CD3. The percentages of CD8^+^PD-1^+^, CD8^+^HLA-DR^+^, and CD8^+^CD154^+^ cells were determined using BV605 anti-CD8, APC-Cy7 anti-HLA-DR, PE anti-CD279 (PD-1), and FITC-anti-CD154. All antibodies were purchased from BD Biosciences (San Jose, CA, USA) and used according to the manufacturer’s directions. Flow cytometry sample acquisition was performed on a LSR Fortessa instrument (BD), and analysis was performed using FlowJo software.

### Cytokine analysis

To identify the cytokines in the ascites fluid, luminex assay was performed using the Milliplex Human Cytokine/Chemokine Magnetic Bead Panel (EMD Millipore, Cat # HCYTMAG-60 K-PX38 and HCYTOMAG-60 K-01) according to manufacturer’s instructions. Briefly, 25 μL of standards, controls, or assay buffer were added to appropriate wells of a pre-washed 96-well plate. Then 25 μL of Serum Matrix buffer were added to standards and control wells, 25ul of samples were added to assay buffer wells, and 25 μL of premixed beads were then added to each well. The plate was mix carefully and sealed with a plate sealer. The plate was then wrapped in foil and incubated on a plate shaker overnight at 4 °C. On the next day, the plate was washed 2 times on a plate washer. Detection antibodies (25 μL) were added to each well. The plate was incubated on a plate shaker for 1 h at room temperature. 25 μL of Streptavidin-Phycoerythrin was added to each well. The plate was then incubated on a plate shaker for 30 min at room temperature, followed by 2 washes. 150 μL of sheath fluid was added to each well and the plate was incubated on a plate shaker for 5 min at room temperature. Lastly, the plate was read on a Bio-Rad Bio-Plex flow cytometer (University of Chicago, Cytometry Core) and the data was analyzed using Bio-Plex software. Reported ratios are the calculated concentration observed for the sample divided by the calculated concentration observed for a healthy PBMC sample control stimulated with phorbol myristate acetate (PMA) and ionomycin.

## Additional file

Additional file 1: Supplementary Table 1: Ratio of ascites-to-stimulated PBMC control values for cytokine arrayanalytes. (XLSX 9 kb)
